# Effects of foot intrinsic muscle dynamic stretching on balance, gait parameters, and dynamic gait index in patients with chronic stroke: A randomized controlled study (CONSORT)

**DOI:** 10.1097/MD.0000000000041507

**Published:** 2025-02-21

**Authors:** Younghwan Kwag, Donghwan Park

**Affiliations:** a Department of Rehabilitation Center, Changwon Hanmaeum Hospital, Changwon, Republic of Korea; b Department of Physical Therapy, Graduate School, Kyungnam University, Changwon, Republic of Korea.

**Keywords:** foot intrinsic muscle stretching, gait, lunge exercise, stroke

## Abstract

**Background:**

Foot intrinsic muscle dynamic stretching intervention can correct balance ability and induce a change in spatiotemporal parameters gait ability. Our objective was to compare the effects of a 4-week program of foot intrinsic muscle dynamic stretching with those of lunge exercise on static balance, dynamic balance, gait parameters (velocity, cadence, step length, and stride length), and the dynamic gait index (DGI) in chronic stroke patients.

**Methods:**

The participants were randomized to either the foot intrinsic muscle dynamic stretching (n = 10) or standard lunge exercise (n = 10) groups. Both groups performed 3 sets of lunge exercises 5 times per week for 4 weeks. Each set comprised 10 repetitions. Static and dynamic balance, gait parameters, and the DGI were measured after 4 weeks of training.

**Results:**

After 4 weeks of training, the foot intrinsic muscle dynamic stretching group showed significant improvement in all outcome measures compared with the baseline (*P* < .05). Furthermore, timed up and go, velocity, step length, stride length, and DGI showed greater improvement in the foot intrinsic muscle dynamic stretching group than in the standard lunge exercise group (*P* < .05).

**Conclusions:**

This study demonstrated that foot intrinsic muscle dynamic stretching training improved dynamic balance, velocity, step lengths, stride length, and DGI in patients with chronic stroke.

## 1. Introduction

Most patients with stroke experience hemiplegia, which leads to impaired gait and balance.^[1]^ Abnormal muscle tone characteristics in patients with stroke weaken the ankle dorsiflexors and cause stiffening of the plantar flexors.^[2]^ This combination of abnormal muscle tone and hemiparesis results in gait dysfunction.^[3]^ Compared to healthy adults, stroke patients exhibit decreased velocity, cadence, stride length, and step length, along with increased time spent in the double-support and stance phases.^[4]^ Additionally, balance disorders are among the most prevalent issues following stroke and have been identified as contributing factors to falls in these patients.^[5]^ Impairment of gait and balance has been reported to negatively impact the performance of activities of daily living in stroke patients, and rehabilitation therapy for gait and balance plays a crucial role in restoring function and providing exercise programs to strengthen muscles, thereby facilitating a return to daily life.^[6,7]^

Lunge exercises are effective for muscle training in areas such as the quadriceps and gluteus maximus muscles.^[8]^ Stepping forward and bending the knees are high-intensity movements that enhance muscle strength by placing significant tension on the hamstrings.^[9]^ Song and Yoo^[10]^ validated the positive effects of lunge exercises on balance and walking capacity during recovery. Ankle joint contracture in stroke patients results in the shortening of the soleus muscle.^[11]^ Additionally, muscle shortening causes mechanical changes in the joints, necessitating joint mobilization and stretching treatments to increase joint range of motion and improve walking ability.^[12]^ Four weeks of strengthening and stretching exercises performed 3 times per day in patients with plantar fasciitis resulted in increased cadence, stride length, and velocity.^[13]^ Stretching, muscle activation, and joint mobilization exercises can be performed through the lunges.^[14]^

Stretching of intrinsic foot muscles can be facilitated by lunge exercises, which concurrently enhance foot flexibility and strength. Stretching is believed to improve athletic performance and prevent injury,^[15]^ whereas strength and power training may provide neuromuscular improvements.^[16,17]^ Furthermore, intrinsic foot muscles influence the ability to generate forward propulsion from one stride to the next, highlighting their role in gait.^[18]^ Cho and Park^[19]^ applied active stretching for 3 days per week, 15 minutes per day, for 6 weeks in stroke patients and found that foot stretching increased cadence, velocity, and stride length. Additionally, Lynn vonGaza^[20]^ applied stretching to the plantar fascia and intrinsic muscles of the foot while enhancing gastrocnemius strength and range of motion in the weight-bearing leg. Finally, static and dynamic stretching of the ankle muscles positively affected postural control during single leg standing, and repetitive ankle stretching improved the biomechanical properties of the calf muscles, tendons, and ankles in patients with stroke, thereby enhancing balance and gait.^[12,21]^

Previous studies have demonstrated that lunge exercises and stretching of the intrinsic foot muscles improve gait and balance; however, the effects of combined lunge exercises and foot intrinsic muscle stretching on balance and gait in patients with chronic stroke have not been investigated. Therefore, this study aimed to examine the effects of dynamic stretching of intrinsic foot muscles on gait and balance in patients with chronic stroke.

## 2. Methods

### 2.1. Study design and participants

This study followed a randomized controlled trial design. Initially, a pilot test involving 6 volunteers was conducted, with each group consisting of 3 participants: 1 group for foot intrinsic muscle dynamic stretching, and the other for standard lunge exercises. The outcomes of this pilot test were then used to perform a power analysis to determine the necessary sample size to achieve a significance level of α = 0.05, power of 0.8, and an effect size of 1.12. The results of the power analysis indicated that 10 patients were required for each study group. G*Power version 3.1.2 software (Franz Faul, University of Kiel, Kiel, Germany) was used for the power analyses.

The inclusion criteria were as follows: (1) confirmation of hemiplegia resulting from stroke, at least 6 months after the stroke; (2) ability to independently walk a distance of 10 m without any assistive devices; (3) a score of 24 or higher on the Korean mini-mental state examination (K-MMSE); (4) ability to perform a lunge independently, placing weight on the affected side; (5) an ankle joint modified Ashworth scale reading between 1 and 2; and (6) stable vital signs. The exclusion criteria were as follows: (1) medical history of botulinum toxin injections in the lower extremities; (2) medications that may affect balance or gait; and (3) susceptibility to falling, indicated by a Berg balance scale score of 20 or below.

This randomized controlled trial was approved by the Institutional Review Board of Changwon Hanmaeum Hospital (approval number: H2023-003). The trial was registered online with the Clinical Research Information Service (ID no. KCT0009017). Before their participation, all patients provided written informed consent to take part in the study. Initial data were collected for each participant, including sex, age, weight, height, duration since stroke occurrence, stroke type, hemiplegic side, and K-MMSE score.

### 2.2. Interventions and procedures

**Figure 1.**
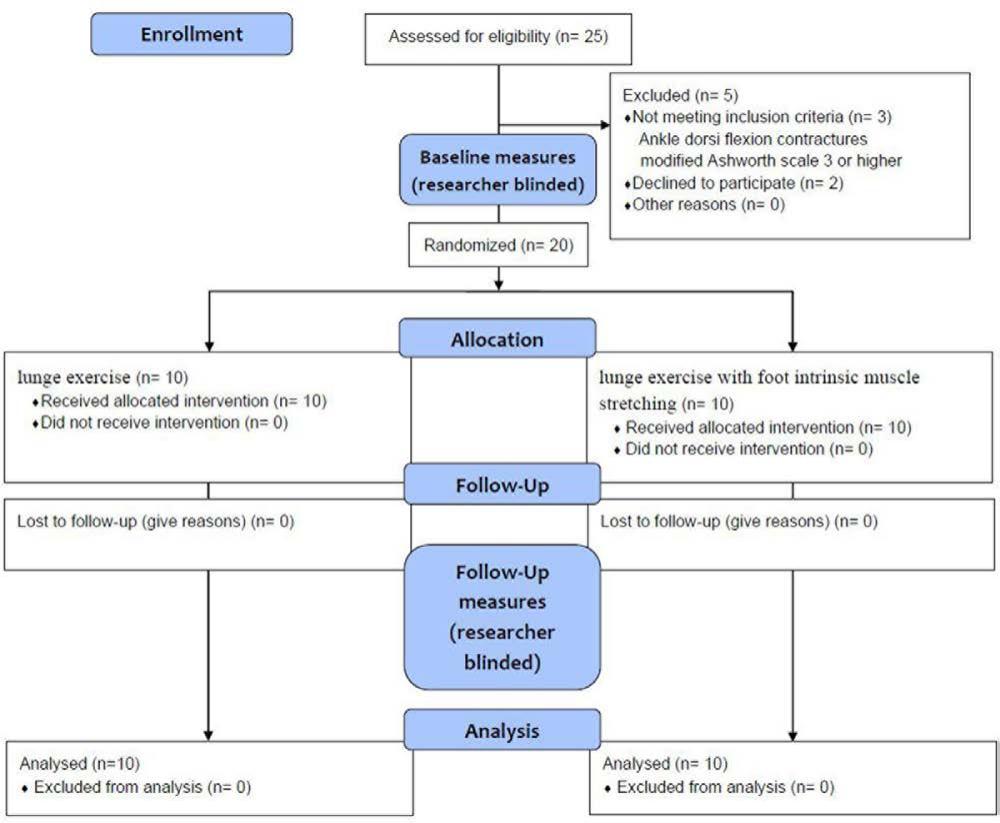
The CONSORT 2010 flow diagram of the experimental protocol.

The principal investigator administered the intervention. The participants were randomized into either the foot intrinsic muscle dynamic stretching group (n = 10) or the standard lunge exercise group (n = 10) using an online randomization program (https://www.randomizer.org/). Patient characteristics and all outcome measures were assessed on the first day of the study and the day after the 4-week intervention by physical therapists with practical clinical experience who were blinded to the group allocation (Fig. 1). Outcome measures included static and dynamic balance, affected-side velocity, cadence, step length, stride length, and dynamic gait index (DGI). At the start of the trial, participants in both groups were familiarized with either foot intrinsic muscle dynamic stretching or standard lunge exercises, depending on their randomization. This familiarization was implemented for approximately 15 minutes per day over 1 to 3 days, depending on the participant. The familiarization period was concluded when the participant demonstrated the ability to perform either foot intrinsic muscle dynamic stretching or standard lunge exercise. Both groups engaged in 3 rounds of lunge exercises per day, 5 days a week, for 4 weeks, with each set comprising 10 repetitions. Throughout the trial, all participants underwent the same rehabilitative training for gait and lower limb function in accordance with the daily inpatient treatment schedule. All participants received standard rehabilitation therapy for 30 minutes per session, with the amount and type of rehabilitation therapy based on a standardized protocol to maintain consistency between the participants in both groups. The initial 5 minutes were allocated to active and passive range of motion (ROM) exercises for the lower limbs of the affected side. The next 15 minutes were spent on weight-bearing training while sitting and standing, followed by 10 minutes of walking. Standard lunge exercises and foot intrinsic muscle dynamic stretching were performed for 4 weeks at the same location under the supervision of the principal investigator to ensure consistent protocol adherence and safety of all enrolled participants.

#### 2.2.1. Standard lunge exercises

The patients were instructed to extend the knee forward until the soleus muscle of the front leg was adequately stretched, ensuring that they avoided any discomfort or pain. In this stretched position, the patients-maintained resistance without lifting their heels for 10 seconds. Subsequently, they returned to their initial position. This lunge exercise routine was repeated 10 times, with 10-second breaks in between.^[22]^ If patients required assistance, they were instructed to grasp the safety lightly.

#### 2.2.2. Foot intrinsic muscle dynamic stretching

**Figure 2.**
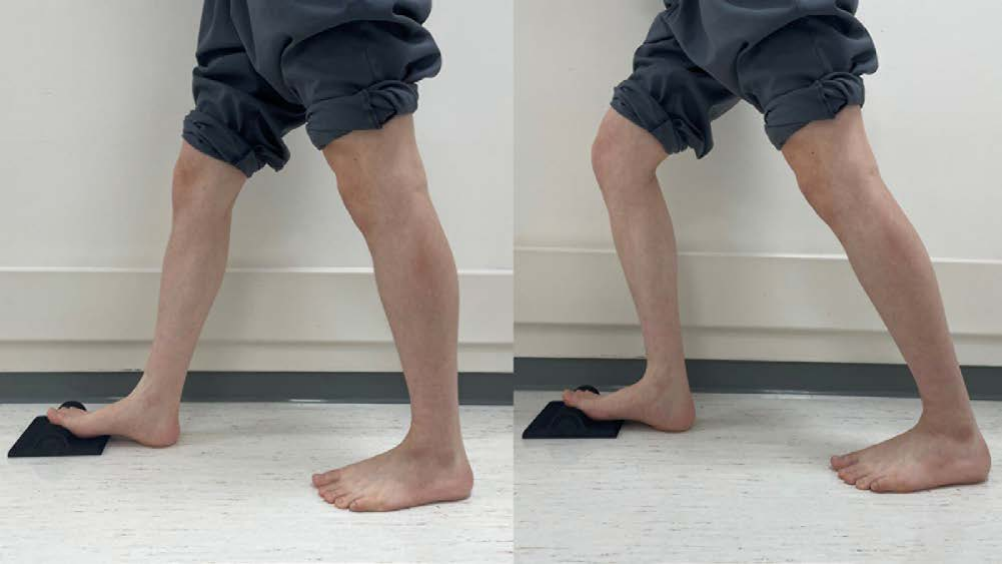
Foot intrinsic muscle dynamic stretching (left side: start position, right side: end position).

All procedures were identical to those of standard lunge exercises, except for the inclusion of a foot intrinsic muscle stretching device. The device used was the Fasciitis Fighter (Fasciitis Fighter Pty Ltd., Australia), and the patients stood on the affected side, placing their great toe on the device. The participants were instructed to keep the back of the knee straight and bend the knee on the affected side, shifting their weight forward until they felt a stretch in the plantar fascia and foot intrinsic muscles (Fig. 2). This exercise was repeated 10 times, with 10-second breaks in between.^[23]^ If patients required assistance, they were instructed to grasp the safety lightly.

### 2.3. Outcome measures

#### 2.3.1. Static balance ability

The static balance ability test was performed before and after treatment using K-FORCE (KINVENT, Montpellier, France), a product range developed by KINVENT for assessing and rehabilitating balance using precise dynamometry instruments. In this study, K-FORCE Plates were used to evaluate balance. The K-FORCE Plates consist of 2 platforms, each containing 2 built-in dynamometers. Measurements were conducted 3 times for 30 seconds, and the average reading was used for data analysis. A stable center of pressure indicates a good static balance.^[24]^

#### 2.3.2. Timed up and go

The timed up and go (TUG) test is commonly used to assess dynamic balance in patients with stroke. This test measures the duration required for an individual to stand up from a chair with armrests, walk 3 m, navigate an obstacle, return to the chair, and sit down again. The TUG test showed remarkable consistency in repeated assessments, with a high intraclass correlation coefficient of 0.95.^[25]^ Measurements were performed thrice, and the average values were used for the final analysis.

#### 2.3.3. Gait parameters

Spatiotemporal gait parameters, including velocity, cadence, stride length, and step length were evaluated using the GAITRite system (CIR Systems, Easton, PA). This system is known for its high dependability in evaluating most aspects of walking, with a reliability index of approximately 0.88 and 0.91.^[26]^ Each participant was instructed to stand at the beginning of the gait mat and to walk comfortably across it until the other end was reached. Gait parameters were measured 3 times and averaged for the final analysis.

#### 2.3.4. Dynamic gait index

The DGI includes 8 specific gait tasks: gait level surface, change in gait speed, gait with horizontal head turns, gait with vertical head turns, gait and pivot turn, step-over obstacles, step-around obstacles, and stairs. These tasks are relevant to both community- and home-based ambulations. Performance levels were categorized into 3 grades using a 4-point scale (normal, 3 points; minor impairment, 2 points; moderate impairment, 1 point; and severe impairment, 0 points) as an assessment tool. The DGI has shown a reliability and validity of 0.96 in research involving older individuals.^[27]^

### 2.4. Statistical analysis

IBM SPSS software (version 27.0; SPSS Inc., Chicago, IL) was used for all the statistical analyses. The normality of the data distribution was examined using the Shapiro–Wilk test. Descriptive data are presented as mean ± standard deviation. After the intervention, independent *t*-tests were used to compare between-group means and paired *t*-tests were used to compare within-group means. Statistical significance was set at *P* < .05.

## 3. Results

### 3.1. Clinical information of the patients with stroke

The baseline characteristics of all the included patients in both groups are summarized in Table 1. No significant baseline differences in patient characteristics such as age, Gender, Hemiplegic side, poststroke duration, K-MMSE, modified Barthel index, or Berg balance scale were detected between the 2 groups (Table 1).

**Table 1:** Clinical information of the patients with stroke.

Characteristics	Lunge exercise (n = 10)	Foot intrinsic muscle dynamic stretching (n = 10)	*P*
Age (yr)	58.9 ± 6.8	50.6 ± 9.2	.088
Gender (male/female)	7/3	4/6	.398
Hemiplegic side (left/right)	6/4	5/5	.548
Poststroke duration (mo)	8.0 ± 2.2	7.8 ± 1.8	.796
K-MMSE	24.3 ± 3.1	25.1 ± 3.6	.565
MBI	63.6 ± 16.0	70.2 ± 19.8	.424
BBS	28.4 ± 8.0	036.7 ± 11.0	.163

BBS = Berg balance scale, K-MMSE = Korean mini-mental state examination, MBI = modified Barthel index; values are expressed as mean ± standard deviation or frequency.

### 3.2. Changes in the balance in each group

The balance ability results are presented in Table 2. No significant differences were observed in any of the baseline values between the 2 groups before treatment. Outcome measures showed significantly improved posttest scores compared to pretest scores in both groups (*P* < .05). Static balance after treatment did not differ significantly between the 2 groups (*P* = .128). However, the TUG score (*P* = .009) significantly improved in the foot intrinsic muscle dynamic stretching group compared with that in the standard lunge exercise group.

**Table 2:** Changes in the balance in each group (n = 20).

Parameters	Lunge exercise (n = 10)		Foot intrinsic muscle dynamic stretching (n = 10)		Between groups *P* value
	Pretest	Posttest	Pretest	Posttest	
Static balance (mm)	7.1 ± 3.3	5.5 ± 3.2*	11.4 ± 7.0	7.9 ± 3.3*	.128
TUG (s)	16.5 ± 4.6	14.6 ± 4.6*	12.5 ± 2.2	9.6 ± 2.2*	.009**

TUG = timed up and go; values are expressed as mean ± standard deviation.

* *P* < .05 indicate a significant difference between pre- and posttreatments within the group.

** *P* < .05 indicate a significant difference between the experimental group.

### 3.3. Changes in the gait parameter in each group

The balance ability results are presented in Table 3. No significant differences were observed in any of the baseline values between the 2 groups before treatment. Outcome measures showed significantly improved posttest scores compared to pretest scores in both groups (*P* < .05). Cadence after treatment did not differ significantly between the 2 groups (*P* = .867). However, velocity (*P* = .019), step length (*P* = .002), and stride length (*P* = .007) were significantly increased in the foot intrinsic muscle dynamic stretching group than in the standard lunge exercise group.

**Table 3:** Changes in the gait parameter in each group (n = 20).

Parameters	Lunge exercise (n = 10)		Foot intrinsic muscle dynamic stretching (n = 10)		Between groups *P* value
	Pretest	Posttest	Pretest	Posttest	
Velocity (m/min)	62.8 ± 21.1	87.3 ± 19.7*	78.8 ± 15.0	112.3 ± 23.5*	.019**
Cadence (step/min)	94.4 ± 29.9	118.5 ± 27.9*	96.3 ± 10.8	116.9 ± 12.5*	.867
Step length (cm)	38.5 ± 9.6	43.1 ± 9.5*	51.3 ± 7.5	58.2 ± 8.5*	.002**
Stride length (cm)	81.7 ± 19.9	85.8 ± 19.9*	99.1 ± 11.3	109.1 ± 14.0*	.008**

Values are expressed as mean ± standard deviation.

* *P* < .05 indicate a significant difference between pre- and posttreatments within the group.

** *P* < .05 indicate a significant difference between the experimental group.

### 3.4. Changes in the DGI in each group

The balance ability results are presented in Table 4. No significant differences were observed in any of the baseline values between the 2 groups before treatment. Outcome measures showed significantly improved posttest scores compared to pretest scores in both groups (*P* < .05). After treatment, the DGI score (*P* < .001) significantly improved in the foot intrinsic muscle dynamic stretching group compared with the standard lunge exercise group.

**Table 4:** Changes in the DGI in each group (n = 20).

Parameters	Lunge exercise (n = 10)		Foot intrinsic muscle dynamic stretching (n = 10)		Between groups *P* value
	Pretest	Posttest	Pretest	Posttest	
DGI (point)	15.1 ± 3.9	18.9 ± 3.8*	17.7 ± 3.1	25.3 ± 2.4*	.001**

DGI = dynamic gait index; values are expressed as mean ± standard deviation.

* *P* < .05 indicate a significant difference between pre- and posttreatments within the group.

** *P* < .05 indicate a significant difference between the experimental group.

## 4. Discussion

This study compared the efficacy of 4 weeks of standard lunge exercises and foot intrinsic muscle dynamic stretching interventions in improving static balance ability, TUG, gait, and DGI in patients with stroke. Our findings support our hypothesis that, after 4 weeks of intervention, the foot intrinsic muscle dynamic stretching group demonstrated significant improvements in TUG, velocity, step length, stride length, and DGI compared to the standard lunge exercise group. To the best of our knowledge, this is the first study to demonstrate the benefits of foot intrinsic muscle dynamic stretching and the effects of an easily applicable brace in stroke patients.

Compared with the standard lunge exercise group, the TUG significantly increased in the foot intrinsic muscle dynamic stretching group by 34.4% after 4 weeks of study intervention. Our results are consistent with those of previous studies.^[28,29]^ Wu, Huang, Lee, Liu, Lin, and Chen^[28]^ examined the effects of dynamic-repeated passive ankle joint motion stretching in the standing position for 15 minutes and showed a significant increase in their TUG time, decreasing from 33.7 to 29.1 seconds in patients with chronic stroke. Lee, Lee, Kwon, and Kim^[29]^ reported that the time taken for the TUG test decreased and foot pressure increased in patients with stroke when intrinsic muscle stretching was performed for 6 weeks. Stretching effectively increases ankle ROM and provides better muscle and joint flexibility during static stretching.^[30]^ Additionally, foot and ankle flexibilities are correlated with dynamic balance.^[31]^ Consequently, improved ankle flexibility through foot intrinsic muscle dynamic stretching helps maintain balance. Additionally, postural control to maintain balance during lunge exercise is achieved through complex interactions between the senses and musculoskeletal system and is integrated and modified within the central nervous system in response to environmental changes.^[32]^ Balance training through foot intrinsic muscle dynamic stretching is thought to improve balance ability as repetitive movements transmit somatosensory sensations from the joints or muscle receptors. Based on our results, we recommend dynamic stretching of foot intrinsic muscle as an effective method to improve balance in patients with chronic stroke.

Compared with the standard lunge exercise group, the velocity significantly increased in the foot intrinsic muscle dynamic stretching group by 27.1% after 4 weeks of study intervention. Our results are consistent with those of previous studies.^[33,34]^ Park, Cynn, Yi, Choi, Shim, and Oh^[33]^ examined the effects of calf muscle stretching in patients with chronic stroke who were instructed to sustain the stretch for 20 seconds, perform the stretch 15 times, and rest for 10 seconds between exercises and found that they had improvements in gait speed. Christiansen^[34]^ examined the effects of hip and ankle stretching in older adults and observed improvements in gait speed. In addition, quadriceps strengthening exercises through lunge exercises have been associated with walking speed in older individuals with mobility disability. The intrinsic foot dynamic stretching is accompanied by isometric contraction because it involves stretching of the ankle and intrinsic muscles,^[35]^ and this isometric contraction has been correlated with walking speed.^[36]^ Additionally, increased gastrocnemius flexibility through stretching contributes to increased dorsiflexion.^[37]^ Increased flexibility and isometric contraction are accompanied by a strong force during push-off,^[38]^ which contributes to increased walking speed.^[39]^ In addition, the foot intrinsic muscles most likely contribute to shock absorption and facilitate efficient foot ground force transmission during walking.^[40]^ In summary, foot intrinsic muscle dynamic stretching, accompanied by isometric contraction of the foot and ankle, is believed to enhance ground reaction force, resulting in a robust push-off force and consequently increasing walking speed. Therefore, based on our results, we recommend dynamic stretching of foot intrinsic muscle as an effective method to improve gait speed in patients with chronic stroke.

After completing the study interventions, step length and stride length significantly improved by 53.5% and 59.5%, respectively, in the foot intrinsic muscle dynamic stretching group compared to the standard lunge exercise group. Our results are consistent with those of previous studies.^[19,41]^ Cho and Park^[19]^ studied the effects of active stretching in patients with chronic stroke and found that ankle ROM, cadence, and stride length were significantly increased. Choi and Kim^[41]^ conducted ankle training in older individuals who had fallen and found gait velocity, cadence, step time, cycle time, step length, and stride length in the ankle training group after the intervention. Generally, factors such as neurological control, balance capabilities, and certain physical and physiological traits such as the coordinated movement of the lower limb muscles and lung capacity can have a significant impact on gait.^[42]^ Lunging can effectively strengthen muscles such as the quadriceps and gluteus maximus^[8]^ and stretching exercises can improve step length.^[43]^ Moreover, improving muscle function through regular exercise increases the number of steps taken and gait speed.^[44]^ Therefore, the foot intrinsic muscle dynamic stretching group, which included strength and stretching exercises, contributed to the qualitative improvement in gait compared with the standard lunge exercise group.

Compared with the standard lunge exercise group, the DGI significantly increased in the foot intrinsic muscle dynamic stretching group by 50.0% after 4 weeks of study intervention. Our findings suggest that interventions focusing on lower body strength and stability, such as foot intrinsic muscle dynamic stretching, may have potential implications for dynamic balance and gait performance, as assessed using the DGI. Previous research has shown that exercises targeting similar lower body muscles are effective in improving balance and gait parameters.^[45]^ Improvements in lower body strength and stability from lunge exercises increase dynamic balance and gait abilities,^[46]^ which may be due to factors such as improved proprioception and increased motor control.^[47]^ All these factors are essential for maintaining stability and control during walking.^[48]^ Furthermore, our findings align with those of previous studies, indicating that exercise programs incorporating lower body strengthening exercises, balance training, and functional activities have been shown to improve mobility and functional performance in individuals with mobility impairments.^[49–51]^ Therefore, our results indicate that foot intrinsic muscle dynamic stretching may improve the dynamic balance and gait.

Our study had several limitations. First, our results may not be generalizable to all patients with chronic stroke because the participants in our study had mild to moderate physical disabilities. Second, the small sample size resulted in lower statistical power, and the absence of continuous follow-up led to a lack of data on long-term treatment effects. Future research should include a larger sample size, and further studies are needed to confirm the persistence of the treatment effect over time. Nevertheless, this study is the first to investigate the effects of foot intrinsic muscle dynamic stretching on the dynamic balance and gait parameters in patients with chronic stroke. Therefore, this study provides a foundation for further research on foot intrinsic muscle dynamic stretching aimed at improving gait function. This study is clinically significant because it demonstrates the effect of foot intrinsic muscle dynamic stretching on gait.

## 5. Conclusion

To the best of our knowledge, this is the first study to evaluate the effects of foot intrinsic muscle dynamic stretching on dynamic balance, velocity, step length, stride length, and the DGI in individuals with chronic stroke. The results of this trial provide evidence that foot intrinsic muscle dynamic stretching, in addition to traditional exercises, could be safe and beneficial for improving the walking quality and maintaining balance in individuals with chronic stroke.

## Acknowledgments

The authors express their gratitude to all patients for their trust and help in making this study possible.

## Author contributions

**Conceptualization:** Younghwan Kwag, Donghwan Park.

**Data curation:** Younghwan Kwag.

**Formal analysis:** Younghwan Kwag, Donghwan Park.

**Funding acquisition:** Younghwan Kwag.

**Investigation:** Younghwan Kwag, Donghwan Park.

**Methodology:** Younghwan Kwag, Donghwan Park.

**Writing – original draft:** Younghwan Kwag.

**Writing – review & editing:** Younghwan Kwag, Donghwan Park.
